# Health, Psychological and Demographic Predictors of Depression in People with Fibromyalgia and Osteoarthritis

**DOI:** 10.3390/ijerph19063413

**Published:** 2022-03-14

**Authors:** Angelina Van Dyne, Jason Moy, Kalila Wash, Linda Thompson, Taylor Skow, Scott C. Roesch, Terry Cronan

**Affiliations:** 1 Department of Psychology, San Diego State University, 5500 Campanile Dr, San Diego, CA 92182, USA; avandyne8479@sdsu.edu (A.V.D.); kwash@sdsu.edu (K.W.); tskow7665@sdsu.edu (T.S.); sroesch@sdsu.edu (S.C.R.); 2 Department of Psychology, University of California San Diego, 9500 Gilman Drive, La Jolla, CA 92093, USA; jhmoy@ucsd.edu; 3 Department of Psychology, University of North Texas, 1155 Union Cir, Denton, TX 76203, USA; lindamthompson18@gmail.com

**Keywords:** fibromyalgia, osteoarthritis, depression, health status, self-efficacy, helplessness, demographics

## Abstract

Depression is common in people with fibromyalgia (FM) and osteoarthritis (OA) and has been linked to adverse health outcomes in these conditions. The purpose of this study was to examine differences in predictors of depression among individuals with FM and OA using a range of health, demographic, and psychological variables. Of the total 963 participants, 600 were diagnosed with FM, and 363 with OA. The Quality of Well-Being Scale (QWB) was used to assess health status. The Fibromyalgia Impact Questionnaire (FIQ) and the Arthritis Impact Measurement Scale (AIMS) were used to measure disease-specific impact. Additionally, participants completed self-efficacy and helplessness assessments. Depression was measured using the Center for Epidemiological Studies Scale (CES-D). The results of a moderated linear regression showed that higher depression scores were associated with lower health status and a greater condition impact, especially in the FM group. Self-efficacy and helplessness predicted depression in both groups, but more strongly in FM. White participants with OA were more depressed than their non-White counterparts, while the opposite was true for FM. These findings indicate that improving health status and psychological well-being might alleviate depression in both FM and OA.

## 1. Introduction

In the United States (U.S.), chronic health conditions are a leading cause of death and disability [1], and the prevalence of chronic illness is expected to continue to increase as the general population ages [2]. A recent Milken Institute analysis revealed that in 2016, the total direct and indirect costs of chronic disease were $3.7 trillion dollars, which is almost 20% of the U.S. economy [3].

Fibromyalgia syndrome (FM) is a chronic musculoskeletal pain condition that is often accompanied by fatigue, headaches, sleep disturbances, memory, and mood difficulties [4]. Two to four percent of the U.S. population is affected by FM, and it is more often diagnosed in women than men, with a previously reported 1:9 male to female ratio [5]. Even though it can develop at any age, the highest prevalence was found in the 50–59 age group [6,7]. The etiology of FM is unknown, with no agreed-upon biological markers, and the treatment focus is on symptom management that incorporates drug and non-drug practices [8].

Osteoarthritis (OA) is a chronic degenerative joint disease that occurs when the cartilage between the bones deteriorates. As a result, people with OA often experience joint pain and irritation of adjacent bone tissue [9,10]. In the U.S., OA is the most common form of arthritis and affects approximately 32.5 million people [10]. It is associated with aging, with the highest prevalence in people over 60 years old, while other risk factors include being female, low levels of education, obesity, having a genetic predisposition, and joint overuse or injury [10,11,12].

FM and OA are prevalent chronic pain conditions with high associated health care costs [13,14]. Despite clinical variations and different etiology, OA and FM share common neural pathways involved in pain and tenderness processing, as well as similar central sensitization to pain [15,16]. Furthermore, FM is frequently misdiagnosed as OA because of symptom similarities, such as morning stiffness and muscle pain [17]. Additionally, the treatment of both conditions requires self-management techniques and effective coping strategies [17,18,19]. Comparing these two chronic painful disorders has been suggested as a way to establish the reference point of the illness impact of both FM and OA [20].

As with other chronic conditions, people with FM and OA often experience psychological difficulties and report higher rates of depression than those in the general population [21,22,23,24,25]. To study depression in people with these illnesses, researchers have investigated various health, psychological and demographic predictors of depression [26,27,28,29,30,31].

Using both general and disease-specific metrics, researchers have found that higher impact of the disease predicted higher depression scores in people with OA and FM [32,33,34,35,36]. Specifically, pain and limited functioning were associated with depression in OA and FM [33,34,35]. While individuals with FM consistently reported higher disease impact than individuals with OA [37], it is unknown to what extent changes in health status impact depression in these conditions.

When examining the association between psychological variables and depression, researchers have found that higher levels of self-efficacy and lower levels of helplessness predicted lower depression in people with FM [38,39,40,41]. Although there are fewer studies examining the direct impact of these psychological factors on depression in people with OA, researchers found that self-efficacy predicted favorable post-surgery outcomes and was linked to reduced disability, while surgery and disability were independently associated with depression [42,43,44,45,46,47]. Additionally, Cronan and Bigatti [48] found that women with FM had significantly higher levels of helplessness and depression than women with OA.

Specific demographic characteristics have also been associated with depression in people with FM, such as being female, not married, young, being from a minority group, low socioeconomic status, and low levels of education [30,49]. Several demographic variables have been shown to predict depression in people with OA. For example, younger adults with OA reported greater depression than older adults [50]. Sale et al. [27] reported that being female predicted higher levels of depression in a sample of 1227 OA patients, even after controlling for negative life events.

Among those with FM, concurrent depression negatively impacted quality of life and resulted in significantly higher health care costs than those with FM who were not depressed [51,52]. At the same time, in people with OA, concurrent depression can adversely affect surgical outcomes, increase drug prescription and the use of health care services, as well as decrease adherence to a treatment regimen [53].

Even though researchers found that patients with FM reported greater depressive symptomatology than OA patients [26,29,49], fewer studies have compared predictors of depression in people with FM and OA [54]. In our lab, independent studies were conducted in which the effects of social support and education intervention were investigated for people with OA and FM. However, the important question of whether the predictors of depression were different for these two populations, with data gathered at baseline, have not been addressed. Given the burden of depression on people with FM and OA and the shared similarities between the two conditions, determining whether the predictors of depression differ among people with FM and OA may assist in the development of more effective treatment strategies and more timely interventions, determine whether the interventions should be different for each condition, and elucidate the mechanisms of depression among those with OA and FM.

The purpose of the present study was to investigate whether the predictors for depression differed for people with OA and FM. The predictors included demographics variables (i.e., age, gender, education, ethnicity, and income), health variables (i.e., quality of well-being, Body Mass Index (BMI), and disease-specific health), and psychological variables (i.e., helplessness and self-efficacy).

## 2. Materials and Methods

### 2.1. Participants

Of the total 963 participants, 600 had an FM diagnosis, and 363 had an OA diagnosis. The data were taken at the baseline assessments. The studies were approved by the Institutional Review Board of San Diego State University (protocol numbers 89-06-188FC and 95-09-293FC with approval dates of 12 September 2014 and 30 June 2015, respectively). The mean age of patients with OA was M = 69.21 (SD = 5.63), and the mean age of participants with FM was M = 53.92 (SD = 11.45). The majority of participants in both groups were White (85.0%-FM; 92.3%-OA), female (95.5%-FM; 64.2%-OA) and completed at least some college. Informed consent was obtained from all participants involved in this study before participating in the research.

### 2.2. Measures

#### 2.2.1. Demographic Variables

Age, gender, ethnicity (minority versus non-minority), highest level of education, and family income were assessed through a demographic questionnaire.

#### 2.2.2. Health Status

The Quality of Well-Being Scale (QWB) was used to measure general health status [55]. The QWB scale was administered by a trained research assistant. The scale is composed of four weighted subscales (symptom complex, mobility, physical activity, and social activity) that combine preference-weighted measures of symptoms and functioning to calculate a numerical value to represent well-being. The numeric value ranges from 0 (death) to 1 (optimal asymptomatic functioning). The validity of the QWB scale has been demonstrated across various chronic illnesses, including arthritis [56] and FM [37]. Reliability has also been demonstrated [57]. Internal consistency reliability is not available because of the nature of the QWB scale’s measuring approach [58].

#### 2.2.3. Helplessness

Participants’ perceived helplessness in coping with OA or FM was measured with the Arthritis Helplessness Index (AHI) questionnaire. The scale was developed for assessing the impact of arthritis, and it was adapted for FM by replacing the word “arthritis” with “fibromyalgia.” The scale consists of 11 items, for which participants are asked to use a six-point scale ranging from 1 (strongly disagree) to 6 (strongly agree) to indicate how much they agree or disagree with each statement. Items were then reverse coded so that higher scores reflected greater helplessness. Adequate internal reliability, test–retest reliability over one year, and construct validity have been demonstrated [59,60]. The coefficient alpha estimate of the questionnaire was reported to be 0.69 [59]. McDonald’s omega was examined to measure internal consistency of the questionnaire in present samples using PROCESS Macro SPSS package (IBM. Chicago, IL, USA). The omega coefficient was 0.77.

#### 2.2.4. Self-Efficacy

The Arthritis Self-Efficacy Scale (ASES) was used to measure perceived self-efficacy for management of and coping with the condition. It was adapted for the FM group by substituting the word “arthritis” for “fibromyalgia” [61]. The ASES consists of 20 items. Participants were asked to rate their certainty that they can manage pain, other symptoms, and perform specific tasks (i.e., walk 100 feet on flat ground in 20 s, walk 10 steps downstairs in 7 s) using a scale ranging from 0 (very uncertain) to 100 (very certain). Higher scores indicate higher self-efficacy. The measure has been demonstrated to have construct validity and reliability [61]. The internal consistency measured by Cronbach’s alpha ranged from 0.76 to 0.89 [61]. McDonald’s omega coefficient was 0.92.

#### 2.2.5. Condition Impact

The Fibromyalgia Impact Questionnaire (FIQ) was used to measure the impact of fibromyalgia on FM participants. The FIQ is a brief 10-item self-administered assessment that measures physical functioning, feeling good, work status, pain, fatigue, sleep, stiffness, anxiety, depression, and well-being [62]. The higher the FIQ score, the greater the impact of FM. The FIQ has shown sufficient construct validity and test–retest reliability and it has been widely used in FM research [63,64]. The measurement has demonstrated good internal consistency: the reported Cronbach alpha was 0.82 [65]. FIQ’s internal consistency for the present sample was assessed by McDonald’s omega using PROCESS Macro package in SPSS and was 0.96.

The Arthritis Impact Measurement Scale (AIMS) was used to measure the disease-specific impact on OA participants. The scale consists of nine subscales to assess mobility, physical activity, dexterity, household activities, activities of daily living, anxiety, depression, social activity, and pain [66]. The reliability and validity of the AIMS were acceptable [66,67]. The internal consistency using Cronbach’s alpha exceeded 0.70 for all nine subscales [66]. McDonald’s omega coefficient was 0.67.

#### 2.2.6. Depression

The Center for Epidemiological Studies Scale (CES-D) was used to measure depression levels [68]. The CES-D is a 20-item, self-administered questionnaire that assesses participants’ depression-related symptoms over the past week. Responses were recorded on a 4-point Likert-type scale ranging from 0 (rarely or none of the time) to 3 (most or all of the time). Four items were reverse coded (e.g., I was happy, I enjoyed life, I felt I was just as good as other people, and I felt hopeful about the future). Items were summed to create a total score so that higher scores indicated more depressive symptoms. A cut-off score of 19 was used for chronic illness populations to indicate depression [69]. The scale has been shown to be both reliable and valid [70,71]. The internal consistency of the scale using Cronbach’s alpha was 0.91 [70]. The reliability coefficient of the present study’s CES-D data measured by McDonald’s omega was 0.92.

### 2.3. Procedures

All participants were recruited from two larger studies investigating the effects of social support and education on health care use and health status. Participants were recruited by mass mailing to members of a large health maintenance organization (HMO) in San Diego, California. Additionally, flyers were posted in HMO waiting rooms, email requests to refer qualified patients were sent to HMO physicians, and advertisements were placed in Sunday newspapers. Participants in the OA study were required to be 60 years or older.

Participants in both the FM and OA groups were required to have a physician’s diagnosis prior to the start of this study, which was later confirmed by a review of the participants’ medical records. In addition, for FM participants, during the initial meeting, trained research assistants performed manual tender point exams using the American College of Rheumatology (ACR) diagnostic criteria [72]. To be eligible, FM participants were required to meet the ACR diagnostic criteria. Informed written consent was obtained from all participants prior to their admission to this study. All measures were collected in person. Demographic and medical history information was recorded by a trained research assistant who also administered the health status measure. Participants completed the helplessness, self-efficacy, condition impact, and depression measures as part of a self-administered battery with a research assistant available to answer questions.

## 3. Results

### 3.1. Descriptive Data

Descriptive data are shown in Table 1. Most FM participants were women. OA participants had lower incomes than FM participants. Health status (well-being) scores were lower in the FM group than in the OA group. The OA group had higher self-efficacy scores. Table 2 shows the correlations among all variables.

### 3.2. Test of Hypothesis

A moderated linear regression was used to test the hypothesis that the predictors for depression would differ as a function of type of chronic condition (OA versus FM). Depression was the dependent variable, and the total CES-D score was used. The demographics (i.e., age, gender, education, ethnicity, income), physical (i.e., quality of well-being, BMI, and health status) and psychological (i.e., helplessness and self-efficacy) variables were the independent variables, and the type of chronic condition was the moderator.

Table 3 shows the results for the regression analysis for physical variables. As indicated in Table 3, there were significant interactions between quality of well-being (β = 0.048, *p* < 0.001), condition impact (β = −3.157, *p* < 0.001) and chronic condition type.

**Figure 1:**
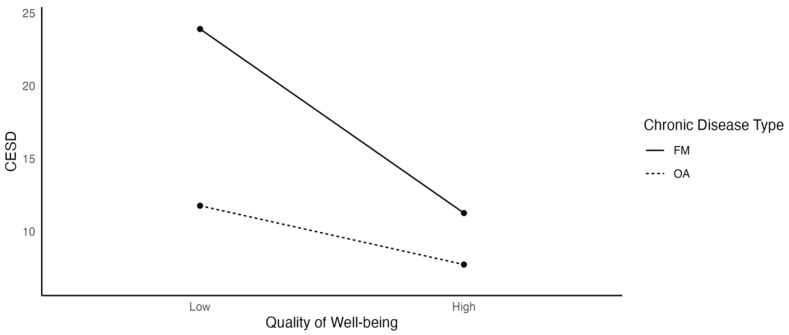
The Effects of Quality of Well-Being on Depression as a Function of Chronic Disease Type.

As shown in Figure 1, depression was negatively associated with quality of well-being, and the relationship was stronger for FM (β = −0.071) than for OA (β = −0.023) participants. Low health status, measured by QWB, was associated with higher depression scores in both groups, but the relationship was more robust in the FM group, indicating a higher sensitivity to changes in health status.

**Figure 2:**
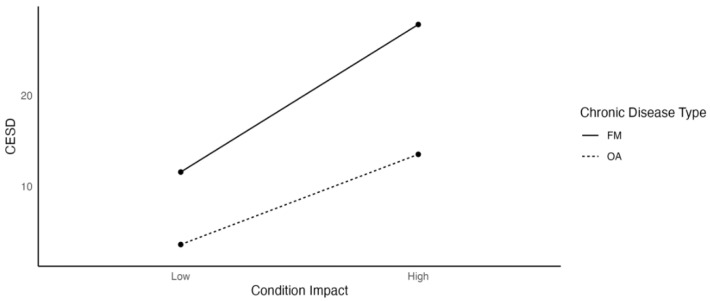
The Effects of Condition Impact on Depression as a Function of Chronic Disease Type.

Figure 2 demonstrates that condition impact was positively related to depression, but the relationship was stronger for FM (β = 8.112) than OA patients (β = 4.965).

We examined the relationships among psychological variables. Table 4 shows that the psychological variables, helplessness (β = −4.217 *p* < 0.001) and self-efficacy (β = 0.108, *p* = 0.003), significantly interacted with chronic condition type.

**Figure 3:**
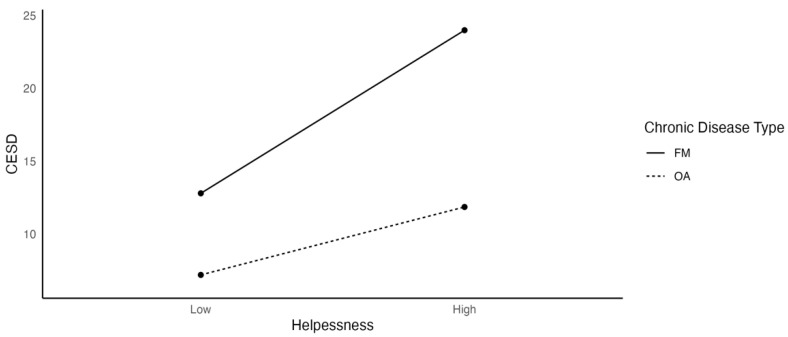
The Effects of Helplessness on Depression as a Function of Chronic Disease Type.

Specifically, Figure 3 shows a positive relationship between helplessness and depression, and that this relationship was stronger for FM (β = 7.228) than for OA patients (β = 3.011). These results indicated that when levels of perceived helplessness were high, depression scores were also higher, and a steeper regression slope indicated that FM patients were particularly sensitive to changes in perceived helplessness.

**Figure 4:**
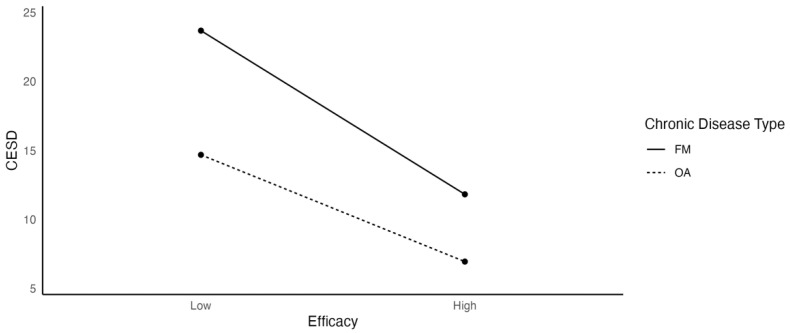
The Effects of Self-Efficacy on Depression as a Function of Chronic Disease Type.

Figure 4 shows that self-efficacy was negatively associated with depression, and the negative relationship was stronger for FM (β = −0.311) than for OA patients (β = −0.203). Higher perceived self-efficacy was associated with lower depression scores especially for participants with FM.

Finally, the results of the moderated linear regression for each of the demographic variables are presented in Table 5.

Among all demographic variables, chronic condition was a significant moderator of education (β = 1.787, *p* = 0.003) and ethnicity (β = 6.475, *p* = 0.006) in predicting depression. Significant interactions were plotted. To further probe the interaction, Table 6 shows slopes estimates for the relationship between depression and individual outcomes among FM and OA patients separately.

**Figure 5:**
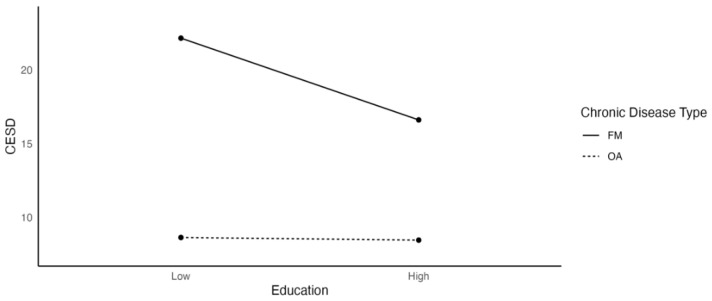
The Effects of Education on Depression as a Function of Chronic Disease Type.

Figure 5 shows that education acted as a buffer for depression among FM (β = −1.846) participants, but not for OA (β = −0.059) participants. Low education in the FM group was associated with higher depression scores, whereas depression scores were stable across education levels among participants with OA.

**Figure 6:**
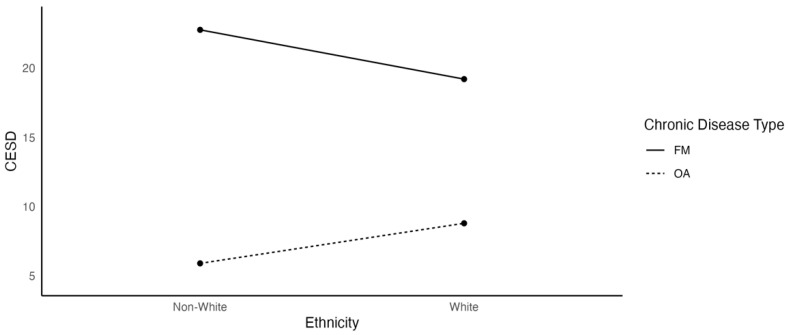
The Effects of Ethnicity on Depression as a Function of Chronic Disease Type.

Figure 6 shows that White OA participants had higher levels of depression than non-White participants, but White FM participants had lower levels of depression than their non-White counterparts. Slope estimates were not computed because ethnicity was a nominal variable.

## 4. Discussion

The purpose of the present study was to determine whether the predictors of depression among people with FM and OA differed. Drawing on the biopsychosocial model of depression, we identified a variety of possible predictors of depression and examined the difference between groups. There were several important findings from the present study.

As hypothesized, both health status and disease impact were significant predictors of depression in the present study. Those with worse health status or disease impact had higher depression scores than those with better health status or less disease impact. Participants with FM also had lower health status and higher disease impact than those with OA. There was a significant interaction such that depression was negatively associated with quality of well-being, and the relationship was stronger for FM than for OA. A significant interaction between group and disease impact indicated that people with FM were more affected by their condition than those with OA. This finding is supported by researchers who found that mental health was strongly contingent upon having a healthy physical condition [73,74], and an association between depression and level of impairment has also been reported among patients with FM and OA [75,76,77,78]. However, FM and OA have distinct clinical features and might impact an individual’s health and depression through different mechanisms. For instance, the pain subscale of the AIMS was found to be strongly associated with depression and, when depression was treated, it resulted in a reduction in pain and disease-related disability in people with OA [21].

On the other hand, the relationship between pain, disability, and depression in FM remains more complicated. Multiple researchers have found that depressed people with FM were more sensitive to pain than non-depressed FM patients [79]. However, others have found that depression did not correlate with pain sensitivity, and researchers did not find any cerebral differences in pain processing between depressed and non-depressed FM patients [77]. They suggested that depressed patients with FM did not experience distorted or augmented pain sensations. However, they found that health status and depression were correlated and, therefore, hypothesized that the mood of individuals with FM might affect the perception of their physical health. In another recent study, pain was not independently associated with quality of life, but other factors such as depression, work status, and activity level were associated with pain [80]. More studies are necessary to clarify the relationship between health status and depression in the FM population.

The findings from the present study indicated that psychological predictors (self-efficacy and helplessness) were associated with depression in people with FM. Specifically, lower levels of self-efficacy and greater helplessness predicted higher depression scores, and this was particularly true in the FM group. These results are consistent with previous research findings indicating that pain conditions may undermine one’s belief in self-efficacy and, as a result, increase depressive symptomatology [81]. The findings from the present study were supported by Van Liew et al. [39], who found that among people with FM, high self-efficacy at baseline predicted fewer symptoms of depression than people with low self-efficacy. They found that individuals who initially had more depressive symptoms were more likely to experience changes in pain intensity at follow-up if their self-efficacy beliefs changed. Buckelew and colleagues [82] proposed that strong self-efficacy beliefs fostered healthier and more consistent coping mechanisms in FM patients that allowed adjustment to the diagnosis and the management of their symptoms.

Among participants in the present samples, high perceived helplessness predicted higher depression scores, particularly for the FM group. These results are not surprising, given that both self-efficacy and helplessness are conceptually similar constructs that describe the opposite ends of the perceived control spectrum. Helplessness was reported to mediate the relationship between pain and depression with FM and was indirectly linked to subjective well-being through its influence on illness uncertainty [41,83]. Individuals with FM were at an increased risk of perceived helplessness because of higher pain, uncontrollability, and the uncertainty of the disease’s etiology and management [79,84]. Some researchers have reported that helplessness can result in the adoption of ineffective coping mechanisms and greater depressive symptomatology [85,86]. However, although this explanation makes intuitive sense, the correlational and cross-sectional nature of these and our study do not allow the establishment of a causal relationship between helplessness and depression [41,85]. It could be argued that feelings of helplessness are direct consequences of depression to which FM patients are predisposed. Future researchers should develop and test interventions to increase a sense of perceived control and reduce helplessness to examine this relationship further.

The results from the present study indicated that participants with FM had significantly higher depression scores than the OA participants across all demographic predictors. Younger participants were significantly more depressed than older participants. In addition, people with lower incomes were more depressed than those with higher incomes. A significant interaction indicated that in the FM group people with lower educational levels had higher depression scores than those with high educational levels; however, there was no difference in the OA group. These findings are consistent with those reported by Güven et al. [30], who found a negative correlation between total years of education and depression in FM participants. However, in the present study, contrary to previous findings [87,88], education did not significantly predict depression in the OA group. A possible, albeit speculative, explanation could be that difficulties related to the unknown etiology and trajectory of FM, as well as the uncertainty associated with the diagnosis, could account for higher depression scores among those with less education. It is also possible that those who are more educated might have an advantage in having more access to high-quality information that provides a buffer for depression. Conversano et al. [89] provide support for this explanation; in a recent meta-analysis, they found that education about methods for self-management improved treatment outcomes for people with FM. A significant interaction indicated that White participants in the OA group were more likely to be depressed than non-White participants. This finding contradicts previous research reports in which minority participants with OA were found to have higher depression levels [90,91]. A possible explanation for this is that ethnic minorities represented only 8% of the OA group, thus creating a statistical limitation. However, in the FM group, White participants were more depressed than non-White participants. Marr et al. [28] reported similar findings. In their study, they found that racial and ethnic minorities experienced greater depression, mood disturbances, pain, and poorer health than their White counterparts. They hypothesized that minority participants experienced greater distress and depression because of pain and poor health.

There were limitations of this study. Because this was correlational research, no conclusions about cause and effect can be drawn. Another significant limitation was the lack of minority participants. Some researchers have reported that there may be a higher prevalence rate of FM among racial minority women [92]. Still, more research is needed to determine whether the prevalence rates vary as a function of ethnicity. Even though similar prevalence rates of OA among different racial and ethnic groups have previously been reported, racial disparities in pain perception and function have been observed among older adults with knee OA [93,94]. Because of the well-established pain-depression link in OA [21], discrepancies in depression and the predictors of it should be examined in racially diverse samples. Future studies focused on minority groups are warranted. In addition, the representation of men with FM was small, which limits the generalizability of the findings to men. The male to female ratio among those with an FM diagnosis in the general population was reported to be 1:9 [5]. However, 95.5 percent of participants in the present FM sample were female; this exceeded the expected prevalence of women in general population. Additionally, while FM and OA are both associated with aging, the findings should be generalized with caution to younger individuals, such as those with juvenile FM or to younger individuals who develop OA as a result of injury. Future research investigating predictors of depression in younger populations with FM and OA is warranted. Furthermore, all the participants came from the same large HMO; there could be differences between those from other health care providers. However, despite these limitations, the number of participants in both the OA and FM groups were large, which increases the likelihood that they represent the populations from which they were drawn.

## 5. Conclusions

In summary, the present study extends current knowledge about the predictors of depression in populations with chronic conditions by comparing two distinct illnesses OA and FM. Our results suggest that while people with chronic conditions are likely to benefit from psychological optimization, treatment for patients with FM should be particularly sensitive to physical symptoms and psychological factors such as self-efficacy, helplessness, and patient education. Thus, intervention studies testing the effects of self-efficacy and helplessness for people with chronic conditions are needed. For people with OA, the predictors of depression had a less pronounced effect than those for people with FM. The reason may be that depression was less prevalent for people with OA and the range of depression scores was more restricted than the range for people with FM. However, depression was related to poorer health outcomes for people with either OA or FM. Taken together, the findings of the present study suggest that health status and levels of self-efficacy and helplessness should be considered in treatment planning to maximize the well-being and health of individuals with either OA or FM.

## Figures and Tables

**Table 1:** Mean and Standard Deviation among Study Variables.

Variable	Mean/Percentage		SD/Range	
	FM	OA	FM	OA
Gender/Women	95.5%	64.2%	-	-
Age	53.918	69.213	11.447	5.626
Ethnicity/White	85.0%	92.3%	-	-
Education ^1^	3.205	3.465	0.914	1.394
Income ^2^	4.712	3.742	2.130	1.768
Well-Being	559.648	642.840	73.469	89.832
Helplessness	3.120	2.612	0.695	0.800
Efficacy	55.592	72.874	17.716	16.124
Body Mass Index (BMI)	29.464	26.958	6.516	5.261
Condition Impact ^3^	0.000	0.000	1.000	1.000

^1^ 1 = grade school, 2 = high school, 3 = some college, 4 = bachelor’s degree, 5 = master’s degree, and 6 = doctorate degree. ^2^ 1 = below $10,000, 2 = $10,001–$20,000, 3 = $20.001–$30,000 4 = $30,001–$40,000, 5 = $50.001–$60,000, 6 = $60.001–$70,000, and 7 = above $70,000/annual. ^3^ Standardized scores.

**Table 2:** Zero-order Correlations among Study Variables.

Variable	1	2	3	4	5	6	7	8	9
1 Gender									
2 Age	−0.250 ***								
3 Ethnicity	−0.036	0.176 ***							
4 Education	−0.157 ***	0.023	0.007						
5 Income	−0.036	−0.255 ***	−0.026	0.249 ***					
6 Well-being	−0.271 ***	0.276 ***	0.055	0.102 *	0.025				
7 Helplessness	0.147 ***	0.221 ***	−0.079	−0.181 ***	−0.042	−0.406 ***			
8 Efficacy	−0.238 ***	0.261 ***	0.081	0.214 ***	0.068	0.547 ***	−0.632 ***		
9 BMI	0.064	−0.163 ***	−0.047	−0.042	−0.020	−0.186 ***	0.134 ***	−0.226 ***	
10 Impact	0.081	−0.134 ***	−0.086	−0.090	−0.135 **	−0.430 ***	0.456 ***	−0.528 ***	0.091

*p < 0.05, ** *p* < 0.01, *** *p* < 0.001.

**Table 3:** Regression Estimates of Physical Variables on Depression.

	Well-Being		Condition Impact		Body Mass Index (BMI)	
	b	*p*-Value	b	*p*-Value	b	*p*-Value
Intercept	59.342	<0.001	19.740	<0.001	18.355	<0.001
Variable	−0.071	<0.001	8.122	<0.001	0.049	0.447
Chronic Condition	−36.256	<0.001	−11.153	<0.001	−10.921	0.002
Variable × Chronic Condition	0.048	<0.001	−3.157	<0.001	−0.009	0.943

**Table 4:** Regression Estimates of Psychological Variables on Depression.

	Helplessness		Efficacy	
	b	*p*-Value	b	*p*-Value
Intercept	−2.812	0.106	37.033	<0.001
Variable	7.228	<0.001	−0.311	<0.001
Chronic Condition	3.509	0.149	−13.648	<0.001
Variable × Chronic Condition	−4.217	<0.001	0.108	0.003

**Table 5:** Regression Estimates of Demographics Variables on Depression.

	Gender		Age		Ethnicity		Education		Income	
	b	*p*-Value	b	*p*-Value	b	*p*-Value	b	*p*-Value	b	*p*-Value
Intercept	19.333	<0.001	30.994	<0.001	22.767	<0.001	25.839	<0.001	23.550	<0.001
Variable	0.426	0.832	−0.209	<0.001	−3.561	0.002	−1.846	<0.001	−0.809	<0.001
Chronic Condition	−12.032	<0.001	−19.546	0.005	−16.878	<0.001	−17.081	<0.001	−13.361	<0.001
Variable × Chronic Condition	1.547	0.502	0.167	0.106	6.457	0.006	1.787	0.003	0.374	0.329

**Table 6:** Slope Estimates for Significant Interactions for FM and OA patients.

	FM		OA	
	b	*p*-Value	b	*p*-Value
Education	−1.846	<0.001	−0.059	0.881
Quality of Well-Being	−0.071	<0.001	−0.023	<0.001
Condition Impact	8.122	<0.001	4.965	<0.001
Helplessness	7.228	<0.001	3.011	<0.001
Efficacy	−0.311	<0.001	−0.203	<0.001

## Data Availability

The data presented in the manuscript are available on request from the corresponding author.
